# The Tuberculin Skin Test versus QuantiFERON TB Gold® in Predicting Tuberculosis Disease in an Adolescent Cohort Study in South Africa

**DOI:** 10.1371/journal.pone.0017984

**Published:** 2011-03-29

**Authors:** Hassan Mahomed, Tony Hawkridge, Suzanne Verver, Deborah Abrahams, Lawrence Geiter, Mark Hatherill, Rodney Ehrlich, Willem A. Hanekom, Gregory D. Hussey

**Affiliations:** 1 South African Tuberculosis Vaccine Initiative, Institute of Infectious Disease and Molecular Medicine, University of Cape Town, Cape Town, South Africa; 2 School of Child and Adolescent Health, University of Cape Town, Cape Town, South Africa; 3 School of Public Health and Family Medicine, University of Cape Town, Cape Town, South Africa; 4 Faculty of Health Sciences, University of Cape Town, Cape Town, South Africa; 5 KNCV Tuberculosis Foundation, The Hague, and CINIMA, Academic Medical Centre, Amsterdam, The Netherlands; 6 Aeras Global TB Vaccine Foundation, Rockville, Maryland, United States of America; McGill University, Canada

## Abstract

**Setting:**

This study was conducted in a high tuberculosis (TB) burden area in Worcester, South Africa, with a notified all TB incidence rate of 1,400/100,000.

**Main Objective:**

To compare the predictive value of a baseline tuberculin skin test (TST) with that of the QuantiFERON TB Gold (In-tube) assay (QFT) for subsequent microbiologically confirmed TB disease among adolescents.

**Methods:**

Adolescents aged 12–18 years were recruited from high schools in the study area. At baseline, blood was drawn for QFT and a TST administered. Participants were followed up for up to 3.8 years for incident TB disease (median 2.4 years).

**Results:**

After exclusions, 5244 (82.4%) of 6,363 adolescents enrolled, were analysed. The TB incidence rate was 0.60 cases per 100 person years (pyrs) (95% CI 0.43–0.82) for baseline TST positive (≥5 mm) participants and 0.64 cases per 100 pyrs (95% CI 0.45–0.87) for baseline QFT positive participants. TB incidence rates were 0.22 per 100 pyrs (0.11–0.39) and 0.22 per 100 pyrs (0.12–0.38) among those with a negative baseline TST and QFT respectively. Sensitivity for incident TB disease was 76.9% for TST and 75.0% for QFT (p = 0.81). Positive predictive value was 1.4% for TST and 1.5% for QFT.

**Conclusion:**

Positive TST and QFT tests were moderately sensitive predictors of progression to microbiologically confirmed TB disease. There was no significant difference in the predictive ability of these tests for TB disease amongst adolescents in this high burden setting. Therefore, these findings do not support use of QFT in preference to TST to predict the risk of TB disease in this study population.

## Introduction

Latent tuberculosis (TB) infection has historically been diagnosed with a tuberculin skin test (TST). However, with this method, cross reactivity with BCG and non tuberculous mycobacteria (NTMs) undermines the specificity of the test[1]. As an alternative to the TST, interferon γ release assays (IGRAs) have been developed which utilise antigens not present in BCG and most non-tuberculous mycobacteria (NTMs)[2]. The advantages of these assays over TSTs are: a second visit for reading of the test is no longer necessary; boosting due to repeated testing is avoided; they have greater specificity for latent TB infection; and they are less prone to the biases encountered when measuring the size of skin reactions, such as digit preference[3]. However, IGRAs are more expensive, need a blood draw and require a sophisticated laboratory.

The QuantiFERON TB Gold (In-tube method) (Cellestis Limited, Carnegie, Victoria, Australia) is one such commercially available assay which has been adopted by many countries as an alternative to TST or as part of a two step approach which uses both tests – a TST is done first and those with a positive test result, have a QuantiFERON done. QuantiFERON TB Gold (In-tube method) (QFT) uses ESAT-6, CFP10 and antigen 7.7 of *mycobacterium tuberculosis* as stimuli to determine if T cells in whole blood are sensitized to such antigens thus indicating prior exposure and/or evidence of latent tuberculosis infection.

The predictive value of a positive tuberculin skin test (TST) for TB disease has been shown in isoniazid prevention trials and contact investigations[4], [5], [6]. When our study was initiated, there was a call to conduct longitudinal studies with the new assays so as to determine their validity in predicting TB disease[7], [8]. Some longitudinal studies have been done to date but the results have been contradictory in that some show a better predictive ability for the IGRAs than for the TST while others show no difference between them[9], [10], [11], [12], [13], [14], [15]. Most of these studies were designed as follow up of contacts of cases diagnosed with TB so it is not clear what the relative value of these tests would be in a general population setting.

We conducted a longitudinal study to determine the predictive value for subsequent TB disease of QFT compared to TST in a large cohort of adolescents in a high burden setting. The baseline characteristics of this adolescent cohort have been published with 55.2% of participants being TST positive (using a 5 mm cutoff) and 50.9% being QFT positive at baseline[16]. Adolescents are currently under investigation as a target group for TB vaccines and the predictive value of interferon gamma release assays for the subsequent onset of TB disease will be helpful in planning clinical trials of novel TB vaccines. Such data are also necessary for policy makers, researchers, and clinicians developing guidelines for the use of such tests in TB control programmes and in clinical practice.

## Methods

### Ethics Statement

Written informed consent was obtained from the parents of participating adolescents and written informed assent obtained from each adolescent. This study was approved by the Faculty of Health Sciences Human Research Ethics Committee, University of Cape Town. Isoniazid prevention therapy (IPT) is not standard of care in South Africa for individuals with latent tuberculosis infection except for infants under the age of 5 and HIV infected persons (National Tuberculosis Management Guidelines, Department of Health, South Africa 2009). Participants with a positive TST or QFT were investigated for TB disease and referred for treatment at public sector facilities if needed.

### Study setting

The study took place at an established TB vaccine trial site in the town of Worcester (and surrounding villages) approximately 100 km from Cape Town. This area has a high burden of TB with a total notified TB incidence rate among all ages of approximately 1,400 per 100,000 population, based on official TB programme data for 2006 [17].

### Study subjects

Adolescents aged 12 to 18 years were recruited from high schools in the study area. They were not participating in a TB vaccine trial at the time of this study.

### Study procedures

emographic data were collected at baseline, as well as data on current and prior household TB contact. At baseline, blood was drawn for QuantiFERON® TB Gold In-tube (Cellestis). A tuberculin skin test was then immediately administered using the Mantoux method on either forearm, using 2 tuberculin units of RT23 (Statens Serum Institut, Denmark). Induration at the TST site was read 48–96 hours later with a ruler or a caliper by trained study personnel. Those with previous or current TB did not have a TST performed, because of the increased risk of severe allergic reactions. The QuantiFERON test (QFT) was performed as recommended by the manufacturers. Any participant who had TB related symptoms, a recent household contact, a positive TST ≥10 mm induration or a positive QFT were referred for two sputum smears. If either or both were sputum positive for acid fast bacilli, the sputums were cultured, a chest x-ray performed and an HIV test done. Those diagnosed with TB were referred to the public health services for evaluation and treatment. About half of participants were allocated to active follow up three monthly and half to passive follow up being seen at baseline and at their two year visit only. At follow up visits, those with new symptoms, a new household contact, a converted TST (>10 mm increase from baseline) or a converted QFT test (change from negative to positive) were investigated for active TB. Investigation for TB included two sputum samples for smear microscopy on two separate occasions. If any single sputum was smear positive, a mycobacterial culture, chest x-ray, and HIV test were performed. In addition, surveillance was conducted at TB clinics and of hospital registers in the area to find any TB cases diagnosed in between visits in all participants. All subjects were scheduled to be seen for a two-year close-out visit unless they were lost to follow up or had died. Due to financial constraints, a small proportion of two-year visits were brought forward at the end of the study. Follow up was therefore continued for a minimum of 22 months. Those completing “two-year” visits were still observed for the occurrence of cases through surveillance of health facility records until the last subject had their final visit, giving a maximum follow up time of 3.8 years. The study took place from 2005 to 2009.

### Analysis

Data were captured in a Microsoft Access database and analysed with STATA version 11.0 (Statacorp, Texas, USA). A TST cutoff of 5 mm was used to define a positive or negative test based on the distribution of TST indurations[16]. Analyses based on the 10 mm and 15 mm cutoffs are also given. A QuantiFERON value of 0.35 international units or more was deemed positive as per manufacturer's specifications. Any participant diagnosed with pulmonary TB based on at least two positive sputum smears or a single positive sputum culture was defined as a case of TB. Incidence rates were calculated by dividing the number of incident cases by the total person time of observation. Observation time was calculated from date of enrolment to study end date except where there was loss to follow up, diagnosis of TB, consent withdrawal or death. When there was loss to follow up, person time of observation was calculated from baseline to a halfway point between the date when last seen and the date of the next scheduled visit when loss to follow up was established. 95% confidence intervals for incidence rates were calculated using Poisson regression. Incidence rate ratios (IRRs) were then calculated and the 95% confidence interval for each IRR was calculated. Exposure to TB was based solely on reported household TB contact at baseline. Reported time between household TB contact and enrolment was calculated by subtracting the year of reported contact from the year of enrolment. The sample size for this study was based on estimation of an incidence rate that would be useful for planning clinical trials of new TB vaccines in adolescents. The sample size analysed would have been sufficient to detect a significant difference in predictive value between the TST positive and QFT positive groups at an incidence rate ratio of 1.54 given an incidence rate of 0.60 per 100 person years in the TST positive group.

## Results

### Study participants

6,363 participants were enrolled at baseline but after exclusion of those with prior or current TB, indeterminate QFT results, or missing QFT or TST results, as described elsewhere[16], 5,244 participants were included in this analysis. 82% of participants completed follow up at two years. Of the 18% of participants who did not complete their two year visits, 8 (0.2%) had died during follow up. More detail on attendance at follow up visits and reasons for visits not being completed is provided separately as Table S1. Mean follow up time was 2.3 years, median 2.4 years and range 1.5 to 46 months (3.8 years). The most common reason for non-participation in this study when a reason was given was fear of blood draws. Important baseline characteristics of the recruited population were as follows: 54.2% female, 56.9% under the age of 16, 93.8% reported having received BCG at birth and 25.4% reported a prior or current household contact. More details on baseline characteristics are laid out in Table 1.

**Table 1:** Demographic profile of study participants analysed (n = 5244).

Category	Numbers (column %)
Gender	
Male	2,402 (45.8%)
Female	2,842 (54.2%)
Age (years)	
>15	2,261 (43.1%)
≤15	2,983 (56.9%)
Racial group	
Black	995 (19.0%)
Mixed race	3,839 (73.2%)
Indian/white	410 (7.8%)
Parent income: classified on at least one parent's income	
≤R4000/month	4,243 (80.9%)
>R4000/month	921 (17.6%)
Unknown	80 (1.5%)
Maternal highest education level	
≤ Primary school	1,510 (28.8%)
≥ High school	2,890 (55.1%)
Unknown:	844 (16.1%)
Paternal highest education level	
≤ Primary school	686 (13.1%)
≥ High school	1,720 (32.8%)
Unknown:	2,838 (54.1%)
BCG reported as being given	
No	46 (0.9%)
Yes	4,917 (93.8%)
Unknown	281 (5.4%)
BCG scar	
Absent	1,490 (28.4%)
Present	2,064 (39.4%)
Unknown (Not sure)	1,690 (32.2%)
Current or prior TB household contact	
Yes	1,332 (25.4%)
No	3,911 (74.6%)
Unknown	1 (0.02%)
Chronic allergy-related conditions e.g. asthma, hay fever, eczema	
Yes	53 (1.0%)
No	5,191 (99.0%)
History of hospitalization within the 6 months prior to enrolment	
Yes	46 (0.9%)
No	5,198 (99.1%)

### Baseline TST and QFT results

There was good agreement at baseline between QFT and TST at the 5 mm (84.8%, kappa 0.70) and 10 mm cutoff (81.4%, kappa 0.63) but not the 15 mm cutoff (64.3%, kappa 0.30) (details described elsewhere[16]).

### Incident cases and rates by TST/QFT status

**Figure 1:**
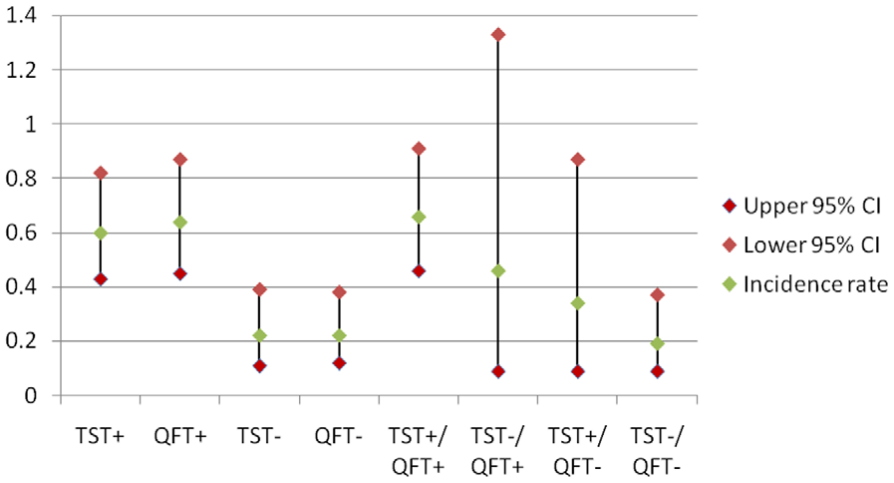
Incidence rates by baseline TST and QFT status. Figure 1 is a chart of incidence rates of tuberculosis per 100 person years of observation by baseline TST and QFT status. Firstly, rates are shown individually for either positive or negative TST or QFT result. Then rates are shown with combined results, either concordant negative or positive TST and QFT results, or discordant negative/positive combinations.

67 participants were diagnosed with TB, of whom 52 met the *a priori* case definition and 42 were culture positive. Percentage agreement between TST and QFT among the 52 cases was 86.5% (kappa 0.63). The incidence rates of TB by baseline QFT and TST status are shown in Table 2 and Figure 1. These show that the TB incidence rate was 0.60 cases per 100 person years (pyrs) (95% confidence interval [CI] 0.43–0.82) for baseline TST positive (≥5 mm) participants and 0.64 cases per 100 pyrs (95% CI 0.45–0.87) for baseline QFT positive participants. There were 7 cases diagnosed within 6 months of enrolment and excluding these changed the rates to 0.53 (95% CI 0.37–0.73) and 0.55 (95% CI 0.38–0.77) cases per 100 pyrs for positive TSTs and QFTs respectively. An additional analysis (not shown in the table) gives an incidence rate of 0.74 per 100 pyrs (95% CI 0.53–1.02) in participants with a baseline TST ≥10 mm (42.2% of participants). There was a significantly higher rate of TB in those with a positive QFT or TST than in those with a corresponding negative test result. The incidence rate ratios (IRRs) were 2.7 (95% confidence interval [CI] 1.4–5.0) for TST (Incidence Rate (IR) TST+/IR TST-) and 2.9 (95% CI 1.6–5.2) for QFT (IR QFT+/IR QFT-). The number of persons that needed to be tested for TST and/or QFT and followed up to predict one TB case over a period of one year ranged from 157 to 520 depending on the test result. The proportion of cases who were test positive and diagnosed with TB within the first year compared to subsequent years was 84.2% versus 72.7% (p = 0.34) for TST and 73.7% versus 75.8% (p = 0.86) for QFT respectively.

**Table 2:** Incidence rates by TST (≥5 mm) and QFT status at baseline.

	N	Incident TB Cases	Person years of follow up	Incidence Rate (per 100 person years, 95%CI)	No of persons followed up per year to detect 1 case
TST+	2,894	40	6651	0.60 (0.43–0.82)	166
QFT+	2,669	39	6137	0.64 (0.45–0.87)	157
TST−	2,350	12	5337	0.22 (0.11–0.39)	444
QFT−	2,575	13	5851	0.22 (0.12–0.38)	450
TST+/QFT+	2,383	36	5480	0.66 (0.46–0.91)	152
TST−/QFT+	286	3	657	0.46 (0.09–1.33)	219
TST+/QFT−	511	4	1171	0.34 (0.09–0.87)	293
TST−/QFT−	2,064	9	4680	0.19 (0.09–0.37)	520
Total*	5,244	52	11988	0.43 (0.32–0.57)	231

*all participants analysed.

### Sensitivity, specificity, positive predictive value and negative predictive value (Table 3)

Approximately three quarters of cases, 76.9% for TST and 75.0% for QFT (p = 0.81), were test positive at baseline. Specificity was less than 50% for both tests: 45.0% for TST and 49.3% for QFT (p<0.01). While this constituted a statistically significant difference, it was not viewed as clinically relevant. The positive predictive value was similarly very low for both (TST 1.4% and QFT 1.5%) and the negative predictive value was equally high for both tests (99.5%).

**Table 3:** Sensitivity, specificity, positive predictive value and negative predictive value of TST and QFT for predicting TB disease.

	TST % (95% CI)	QFT %(95% CI)
Sensitivity	76.9 (63.6–87.5)	75.0 (61.1–86.0)
Specificity	45.0 (43.7–46.4)	49.3 (48.0–50.7)
Positive Predictive value	1.4 (1.0–1.9)	1.5 (1.0–2.0)
Negative predictive value	99.5 (99.1–99.7)	99.5 (99.1–99.7)

### Relationship between time of exposure and risk of disease

In one subset of participants (n = 1328) for whom a history of a household TB contact was reported at baseline, the prevalence of a positive baseline TST and QFT was significantly and negatively associated with time since TB contact exposure (Table 4); i.e. the longer the time since last recalled contact, the lower the proportion with a positive result on either test. Of 20 cases for whom a TB contact prior to enrolment was reported, 15 (75.0%) had had a contact less than 5 years before enrolment and 5 (26.3%), 5 years or more. The proportion positive TST and QFT results among cases who reported recent compared to remote contacts were similar. Rates of disease were higher amongst those more recently exposed than among those exposed ≥5 years or more previously. These differences were not significant since all incidence rate ratios had very wide confidence intervals which included one (Table 5).

**Table 4:** Proportion TST and QFT positive by time since recalled exposure to household TB contact (N = 1,328).

	QFT positive (%)	TST positive (%)
0–4 years (n = 890)	69.4	72.3
5–9 years (n = 235)	65.1	72.8
10–14 years (n = 168)	57.1	67.3
≥15 years (n = 35)	57.1	54.3
Chi squared for trend:	p<0.001	P = 0.036

**Table 5:** Incidence rates by time since recalled exposure to household TB contact.

Time since contact	Numbers reporting contact n = 1,328	Incident cases	Incidence rate per 100 person years	QFT + incident cases	QFT + incidence rate per 100 person years	TST + incident cases	TST + incidence rate per 100 person years
Recent <5 years	890	15	0.74	15	1.07	14	0.95
Remote ≥5 years	438	5	0.49	3	0.48	4	0.56
Incidence rate ratio			1.5		2.2		1.7
(Recent/remote) (95% confidence intervals)			(0.5–5.3)		(0.6–11.9)		(0.5–7.0)

## Discussion

This is the largest study investigating the predictive value of TST versus an IGRA and it is one of few from a high burden setting (Table 6). A positive TST and QFT were both indicative of a higher risk of developing subsequent TB disease, and were equivalent in predicting incident TB disease. While the sensitivity was moderate, specificity and positive predictive values were relatively low, and negative predictive values were high. There was a trend for more recent reported exposure (<5 years) to be associated with a higher risk of disease than more remote exposure but the confidence intervals were very wide.

**Table 6:** A summary of longitudinal studies comparing the predictive value of TSTs and IGRAs.

Authors, Country and Year of publication	Study Type	Number	Population description	Cases	Length of Follow up	Products and TST cutoffs	Baseline prevalence	Cumulative incidence (%) or Incidence rate per 100 person years (pyrs)*
R Diel et al. Germany (2010)	Contact investigation	903	All ages - range 1–62	19	Up to 4 yrs	TST ≥5 mm	63%	3.2%
						TST ≥10 mm	25%	4.8%
						QFT	21%	12.9%
P Hill et al. Gambia (2008)	Contact investigation	2348	All ages	26	2 yrs	TST ≥10 mm	36%	0.9/100 pyrs
						Elispot	28%	0.9/100 pyrs
S Kik et al. The Netherlands (2009)	Contact investigation	339	Immigrants ≥16 years	9	2 yrs	TST ≥10 mm	54%	3.1%
						QFT	55%	2.8%
						T-Spot	63%	3.3%
M Bakir et al. Turkey (2009)	Contact investigation	908	Age ≤16 years	15	2 yrs	TST ≥5 mm	61%	1.7/100 pyrs
						Elispot	42%	2.1/100 pyrs
CC Leung et al. Hong Kong (2010)	Cohort study	308	Adult males with silicosis	17	1–5 yrs	TST ≥5 mm	74%	2.3/100 pyrs
						TST ≥10 mm	66%	2.6/100 pyrs
						T - Spot	66%	3.2/100 pyrs
C Lienhardt et al. Senegal (2010)	Contact investigation	2679	All ages	52	2 yrs	TST ≥5 mm	78%	1.5/100 pyrs
						TST ≥10 mm	65%	1.2/100 pyrs
						TST ≥15 mm	37%	1.6/100 pyrs
						Elispot	65%	1.4/100 pyrs
H del Corral et al. Colombia (2009)	Contact investigation	2052	All ages	37	2–3 yrs	Elisa (IFN-γ responses to CFP-10)	66.3%	0.8/100 pyrs
H Mahomed et al. South Africa	Cohort study	5244	Adolescents aged 12–18	52	2–4 yrs	TST ≥5 mm	55%	0.6/100 pyrs
						TST ≥10 mm	42%	0.7/100 pyrs
						QFT	51%	0.6/100 pyrs

*Incidence rates adjusted to per 100 person years from original articles to enable comparison.

This study is significant in that most of the other longitudinal studies examining this question were based largely on household contacts of TB cases[9], [10], [12], [13], [15], [18], [19], [20] while those using a cohort methodology were much smaller by comparison[11], [14]. The finding in this study of a similar predictive value for QFT and TST accords with that of studies in Turkey, the Gambia, Senegal, Norway and the Netherlands [10], [11], [12], [13], [15], but contradicts the findings of the studies from Germany and Hong Kong[9], [14]. It is thus still unclear whether IGRAs offer better predictive value for subsequent TB disease than the TST. There is increasing evidence that IGRAs perform differently in high burden compared to low burden countries and this may partly explain the differences seen amongst the different studies[21]. The study in The Netherlands was done amongst immigrants and the one in Norway amongst asylum seekers, populations that represent high incidence home countries rather than low incidence host countries[11], [13]. In countries with a high burden of TB disease and therefore exposure to active TB cases, the greater specificity of IGRAs is apparently not useful. Also, where BCG is given at birth as is common in high burden countries, it has been shown that the effect of BCG on the TST is limited after the age of 10 and the TST thus retains its specificity[22]. These tests may therefore perform better in low incidence settings. Therefore, many low incidence countries have shifted to using IGRAs.

Table 6 summarises the studies comparing the predictive value of TST and IGRAs for TB disease and includes the data from this study. There are important differences amongst the studies – different products are compared, different cutoffs are used for TST and different populations have been studied. These design and measurement differences may also explain some of the different results obtained. This limits the degree to which these studies are comparable.

The TB incidence rates in our cohort study are lower than in the contact investigation studies. Contacts are likely to have been more recently exposed whereas in a cohort study, time since exposure will vary. Recent contacts are at higher risk of progressing to disease within the first two to five years of follow up as demonstrated in early studies in the USA which give a 10 year rate of disease of 36.9 per 1000 amongst household contacts compared to a 10 year rate of 6.6 in a cohort of mental patients who were followed up[4]. In the household contact group in this American paper, most cases occurred in the first 5 years of follow up. The kind of cohort study reported in this manuscript helps to quantify risk in a clinical context where time since exposure is often not known.

The lower proportion of TST and QFT positive results in those with a longer reported time between exposure and latent TB infection measurement by TST and QFT indicates waning of responses to tuberculosis antigens over time although this may also be affected by recall bias. When we examined risk of progression to disease in those who reported a prior household contact, there was a trend towards an increased risk in those with recent exposure but this was not significant. This study may have been inadequately powered to detect this difference. Also, one would need to recognize that in this community, the high burden of TB means that exposure is likely to be a common occurrence whether reported or not. A study amongst immigrants to the Netherlands showed that remote exposure is common particularly among those from high burden settings[23].

There was no difference in proportion TST or QFT positive at baseline between cases diagnosed in the first year after enrolment versus those diagnosed afterwards. This may be due to the fact that follow up was not continued for long enough to detect a drop off in risk. The studies reviewed by Ferebee show a sustained high risk in those with a recent contact in the first five years of follow up before the risk starts declining[4].

Despite the higher risk of subsequent TB represented by a positive TST or QFT, the number of people that must be followed up and screened for TB in order to find a single case is substantial, even in this high burden setting where infections and re-infections are common[24]. The cost effectiveness of such population screening would need to be evaluated, including the value of treating test positive persons with isoniazid preventive therapy to prevent the onset of future TB disease. Given that across most studies, more than 95% of persons who are IGRA positive do not progress to TB disease, emphasises the need for biomarkers other than interferon gamma for risk prediction, or a combination of interferon gamma with risk factors (e.g. age, contact history, conversion) to enhance predictive value.

Positive and negative predictive values are dependent on prevalence. Since a low positive predictive value was achieved in a high burden setting, an even lower PPV can be expected in a low burden setting. The findings on sensitivity, specificity, positive and negative predictive value are similar to those in The Netherlands study[13] while the other studies did not describe these. While a positive test is not very helpful in predicting disease, a negative test suggests that risk of progression to disease is low although it does not rule out the possibility entirely.

These results are not representative of adolescents with prior TB since they were excluded from having a TST. Since those with past TB are at higher risk of getting TB again, our incidence rates are probably all underestimated. Since those with past TB are also more likely to be TST and QFT positive, it is unclear how sensitivity, specificity, PPV and NPV would change, had we included those. Also, any of those negative at baseline could have converted during study follow up due to new exposures prior to the onset of TB disease and would be misclassified as test negative. There is no reason to think that this would have occurred differentially between the tests. Those with a positive TST or QFT at baseline were investigated for TB. If diagnosed with TB, they were not included in the 5244 participants analysed in this predictive analysis. Those with a QFT or TST conversion were investigated at follow up and if diagnosed were included in the analysis. However, most cases (>80%) were diagnosed by local health services who would not normally have enquired about TST and QFT results since these are not routinely used in the diagnosis of TB in this age group. Therefore neither incorporation bias nor lack of blinding were felt to be major factors influencing the analysis. While the screening methods for TB at baseline were insensitive for smear negative culture positive TB, a sensitivity analysis which excluded cases diagnosed within six months of enrolment did not appreciably change the main findings showing no difference in the predictive value of TST versus QFT for TB disease. Not all participants completed follow up; if any of these had developed TB, this may have influenced the findings in that rates would have been higher. Finally, since recruitment took place at schools and a substantial proportion (41.8%) did not participate, these results are not representative of all adolescents in this area. None of these limitations are thought to affect the comparison between TST and QFT in this study.

These findings suggest TST and QFT are equally predictive of progression to TB disease in a cohort of adolescents in a high TB burden population and may be used interchangeably. Our results do not support the hypothesis that QFT is superior to TST in its predictive value. These findings should assist policy makers attempting to develop guidelines for IGRA use in high and low TB burden countries. More studies in high burden settings and in adolescents are needed to indicate whether either the TST or QFT may be used as a screening tool in planning TB vaccine trials in adolescents.

## Supporting Information

Table S1 **Flow of visits and reasons for visits not taking place.** This table describes frequency of visits, the number of visits at each time point and the reasons for visits not being completed. It also provides the number for each reason with percentages.
(DOC)
