# Correction: The Tuberculin Skin Test versus QuantiFERON TB Gold® in Predicting Tuberculosis Disease in an Adolescent Cohort Study in South Africa

**DOI:** 10.1371/annotation/b371be66-de38-4ed6-b440-d27c9d7e552c

**Published:** 2011-06-30

**Authors:** Hassan Mahomed, Tony Hawkridge, Suzanne Verver, Deborah Abrahams, Lawrence Geiter, Mark Hatherill, Rodney Ehrlich, Willem A. Hanekom, Gregory D. Hussey

Reference 12 is incorrect. The correct reference 12 is: "Hill PC, Jackson-Sillah DJ, Fox A, Brookes RH, de Jong BC, et al. (2008) Incidence of tuberculosis and the predictive value of ELISPOT and Mantoux tests in Gambian case contacts. PLoS One 3: e1379". 

