# Supplementary material for: The Tuberculin Skin Test versus QuantiFERON TB Gold® in Predicting Tuberculosis Disease in an Adolescent Cohort Study in South Africa
Source: PLoS One. 2011 Mar 29;6(3):e17984. doi: 10.1371/journal.pone.0017984 (PMC3066222; doi:10.1371/journal.pone.0017984)
Supplement: Table S1 — Flow of visits and reasons for visits not taking place. This table describes frequency of visits, the number of visits at each time point and the reasons for visits not being completed. It also provides the number for each reason with percentages. (DOC) [file pone.0017984.s001.doc]

**Table S1: Flow of visits and reasons for visits not taking place.**

Visit Died Other Loss to Withdrew Missed Seen Not Seen^a^ Total

Day follow up Visits

90 1 0 18 12 5 2,765 2,443 5,244

% 0.02 0.00 0.34 0.23 0.10 52.73 46.59 100.00

180 1 0 62 73 29 2,636 2,443 5,244

% 0.02 0.00 1.18 1.39 0.55 50.27 46.59 100.00

270 1 0 69 77 19 2,635 2,443 5,244

% 0.02 0.00 1.32 1.47 0.36 50.25 46.59 100.00

360 1 1 87 159 33 2,520 2,443 5,244

% 0.02 0.00 1.66 3.03 0.63 48.05 46.59 100.00

450 2 1 90 162 23 2,523 2,443 5,244

% 0.04 0.02 1.72 3.09 0.44 48.11 46.59 100.00

540 2 1 133 261 52 2,352 2,443 5,244

% 0.04 0.02 2.54 4.98 0.99 44.85 46.59 100.00

630 2 1 146 282 150 2,220 2,443 5,244

% 0.04 0.02 2.78 5.38 2.86 42.33 46.59 100.00

720 8 0 409 537 0 4,290 0 5,244

% 0.15 0.00 7.80 10.24 0.00 81.81 0.00 100.00

^a^ Roughly half of participants were followed up three monthly and half were seen at baseline and two year visit. These figures reflect the group only seen at baseline and day 720 visits. This component of this study will be dealt with in a separate manuscript and does not have any bearing on the analysis shown in this manuscript.
